# Editorial: Advanced genomic techniques for congenital adrenal hyperplasia

**DOI:** 10.3389/fendo.2026.1882809

**Published:** 2026-06-17

**Authors:** Semra Çetinkaya, Henrik Falhammar

**Affiliations:** 1 Department of Pediatric Endocrinology, Dr. Sami Ulus Obstetrics and Gynecology, Child Health, University of Health Sciences, Istanbul, Türkiye; 2 Diseases Training and Research Hospital, Clinic of Child Health and Diseases, Ankara, Türkiye; 3 Ankara University, Institute of Health Sciences, Ankara, Türkiye; 4 Department of Molecular Medicine and Surgery, Karolinska Institutet, Stockholm, Sweden; 5 Department of Endocrinology, Karolinska University Hospital, Stockholm, Sweden

**Keywords:** congenital adrenal hyperplasia, follow up, genomic techniques, new technique, treatment

This Research Topic is designed to address advanced genomic techniques related to congenital adrenal hyperplasia (CAH), to examine the complete DNA sequence (genome), the interaction of genes with each other and the environment, to reveal genetic complexities, and to broaden our horizons regarding innovative treatment strategies tailored to individual needs. The most common type of CAH is due to 21-hydroxylase deficiency (21OHD) (1, 2). The *CYP21A2* locus contains a functional gene and a pseudogene; the complexity of the *CYP21A2* locus and the genetic heterogeneity of the disease cause diagnostic challenges (3, 4).

This Research Topic includes two review articles and two original research articles:

The review by de Miranda et al. discusses the evolution of molecular diagnostic strategies for 21OHD, highlighting strong population-based phenotype-genotype associations, but also including rare cases that do not show a phenotype-genotype association. It has been noted that classic diagnostic methods cannot resolve misalignment artifacts as well as complex structural rearrangements, and its performance is limited in the *RCCX* region, therefore, advanced genomic techniques such as Sanger sequencing, multiplex ligation-dependent probe amplification (MLPA), Next Generation Sequencing, and long-read sequencing (LRS) have been developed. In particular, it has been emphasized that the LRS method, the newest diagnostic assessment which is a single-platform technology that resolves the gene and pseudogene module, can accurately detect all variant classes (precise gene-pseudogene discrimination), performs accurate breakpoint mapping, and can directly determine the cis/trans phase. LRS has been proposed as the future reference method for high-resolution genotyping, with the potential to transform newborn screening algorithms, reduce false positives, reshape clinical practice, enhance genetic counseling, and support personalized treatment strategies.

In the review article by Graves et al. discuss genome editing of the *CYP21A2* gene (the gene responsible for 21OHD) and the possibility of achieving a permanent cure. The structure of this gene is complex. In genome editing, reaching adrenocortical progenitor cells can be difficult. Gene transfer vectors may include adenoviral/retroviral/adeno-associated virus vectors or non-viral vectors. Gene transfer strategies include gene insertion, gene transfer with transposases (The biggest clinical limitation of gene therapies with adenovirus-related vectors is the presence of pre-existing or post-treatment neutralizing antibodies. Transposase systems offer an alternative method that completely solves this problem), gene editing, and CRISPR/Cas. In gene insertion, gene editing has become relevant due to the loss of the transcriptionally active episome during cellular replication. Gene editing involves a range of technologies based on meganucleases, zinc finger nucleases, transcription activator-like effector nucleases, and programmable nucleases. Prime editors used in gene editing include homology-independent targeted integration, targeted transposition, and base editors. Base editors are presented as an efficient method allowing for programmable repair or correction of point variants without double-stranded DNA cutting, without the need for donor templates, and without reliance on unpredictable DNA repair. However, prime editing is performed using CRISPR-based technology that allows for “search and replace” modification of the genome. The prime editor protein (a combination of Cas9 nicase and reverse transcriptase) inserts targeted DNA alterations without breaking the double-stranded DNA; it is suitable for point variants and small nucleotide sequence insertions, but does not allow the insertion of an entire gene coding sequence. Off-target DNA cutting with CRISPR/Cas9 can lead to disruption of important genes, insertion mutations, or other undesirable genomic aberrations. This carries potential risks, primarily in oncogenesis, but also in the immunogenicity of producing a protein not previously produced endogenously and the involuntary regulation of germ cells. Gene editing has the potential to provide a powerful and lasting treatment for CAH if the elusive adrenal cortex progenitor cells are targeted. Special variant-specific therapies may be available, but it may not be commercially viable yet.

Kolli et al. evaluated circulating miRNA profiles for the first time in the literature in 37 patients with 21OHD, with a median age of 28 years. Circulating miRNA profiles are dynamic and provide critical information about the development and progression of various diseases such as cancer, neurological and cardiovascular diseases. Previous studies have shown that glucocorticoids regulate miRNA expression, affect glucocorticoid receptor expression and function, reduce glucocorticoid cell sensitivity, increase glucocorticoid resistance, and target inflammatory pathways (5). Understanding the effects of glucocorticoid therapy on miRNAs that regulate various biological processes is important to help us develop treatment strategies and identify new biomarkers that can be used in optimizing treatment. In this study, no significant difference was observed in miRNA expression between high and moderate glucocorticoid doses, and between moderate and physiological glucocorticoid doses. However, some expressed miRNAs (such as miRNA-320a, miRNA-122, miRNA-let-7b, miRNA-let-7i, miRNA-4747, miRNA-3591, and miRNA-4732) showed differences (negatively regulated) depending on the high dose of glucocorticoids. They were found to play a role primarily in cell cycle progression, metabolic dysfunction, circadian rhythms, glucocorticoid receptor signaling, and the regulation of stress-related target genes. As a result of this study, it was determined that miRNAs play important roles in inhibiting cancer cell proliferation, immune regulation, oxidative stress, insulin resistance, or metabolic syndrome according to different glucocorticoid regimens. This study has expanded our understanding of the role of miRNAs in the epigenetic mechanisms underlying adverse outcomes associated with supraphysiological glucocorticoid doses in patients with CAH. It has been suggested that miRNAs could be used as important biomarkers in treatment monitoring.

Sappl et al. conducted a study focusing on CAH-X syndrome in a cohort of 155 patients with CAH. Tenascin-X (TNX) haploinsufficiency is a genomic condition where a pathological variant in one copy of the *TNXB* gene reduces the production of the functional tenascin-X protein by approximately 50%, resulting in a connective tissue disorder often linked to the hypermobile form of Ehlers-Danlos syndrome (EDS). It is frequently associated with CAH in a “contiguous gene syndrome” known as CAH-X. Advanced genomic techniques have been critical in identifying these variants due to the complex, high-homology nature of the *TNXB* gene and its adjacent pseudogene, TNXA (6). In the Munich CAH cohort, 94 patients with salt-wasting phenotype, 44 with simple virilizing phenotype, 12 with non-classical pheontype, 4 with carrier type, and 1 with 11β-hydroxylase deficiency were included. The frequency of CAH-X syndrome was found to be 5%. Cardiac abnormalities such as increased muscle echogenicity, persistent truncus arteriosus, and relaxation disorders, particularly in the legs, were found in patients with CAH-X syndrome. The importance of follow-up for these conditions in these patients with CAH-X is emphasized.

With advanced genomic techniques in CAH, our perspectives on diagnosis, treatment, and follow-up have broadened, and our knowledge of complex genetic structures has increased.
